# Methicillin-resistant *Staphylococcus aureus* Clones, Western Australia

**DOI:** 10.3201/eid1202.050454

**Published:** 2006-02

**Authors:** Geoffrey W. Coombs, Julie C. Pearson, Frances G. O'Brien, Ronan J. Murray, Warren B. Grubb, Keryn J. Christiansen

**Affiliations:** * Royal Perth Hospital, Perth, Western Australia, Australia;; † Curtin University of Technology, Bentley, Western Australia, Australia

**Keywords:** Staphylococcus aureus, epidemic MRSA, community MRSA, multilocus sequence typing, SCCmec, PVL toxin, research

## Abstract

Community-associated methicillin-resistant *Staphylococcus aureus* (MRSA) was first reported in Western Australia in the early 1990s from indigenous peoples living in remote areas. Although a statewide policy of screening all hospital patients and staff who have lived outside the state for MRSA has prevented the establishment of multidrug-resistant epidemic MRSA, the policy has not prevented SCC*mec* type IV and type V MRSA clones from becoming established. Of the 4,099 MRSA isolates analyzed (referred to the Gram-positive Bacteria Typing and Research Unit) from July 2003 to December 2004, 77.5% were community-associated MRSA (CA-MRSA). Using multilocus sequence/staphylococcal chromosome cassette *mec* typing, 22 CA-MRSA clones were characterized. Of these isolates, 55.5% were resistant to >1 non–β-lactam antimicrobial drug. Five Panton-Valentine leukocidin (PVL)–positive CA-MRSA clones were identified. The emergence of multidrug-resistant CA-MRSA clones and the detection of PVL toxin genes in clones previously reported as PVL negative is a major public health concern.

## Abstract 2

The emergence of multiple multidrug-resistant Panton-Valentine leukocidin–positive MRSA clones in the community is a major public health concern.

*Staphylococcus aureus* successfully colonizes humans, contaminates the hospital environment, and has the genetic versatility to acquire resistance to multiple antimicrobial agents. Methicillin-resistant *S. aureus* (MRSA) was first detected soon after the introduction of methicillin in 1960, and isolation rates increased until the early 1970s (*1*). These earlier "classic" MRSA strains were genetically similar to each other and may have evolved from a single clone (*2*). In 1976, the first outbreak of gentamicin-resistant MRSA in Australia (*3*) was reported, and by 1981 extensive outbreaks occurred in several countries. In 1985, it became evident that these "modern" strains of MRSA carried epidemic potential not possessed by MRSA isolated in the 1960s and early 1970s and that they were genetically different from the earlier classic MRSA (*4*). Since 1990, international and intercontinental spread of MRSA (known as epidemic MRSA or EMRSA) has increased. In 2002, Enright et al., using multilocus sequence typing (MLST) combined with staphylococcal chromosome cassette *mec* (SCC*mec*) typing, established that relatively few major EMRSA clones existed (*5*). These clones emerged either as descendants of preexisting EMRSA clones or by horizontal transfer of the *mec* determinants into methicillin-susceptible *S. aureus*.

EMRSA became endemic in hospitals in eastern Australian states (New South Wales, Victoria, and Queensland) in the late 1980s and 1990s, with some spread to hospitals in South Australia, the Northern Territory, and Tasmania (*6*). However, a statewide MRSA policy, introduced in 1982, prevented these strains from becoming established in Western Australia (WA) hospitals. This policy required MRSA screening of anyone who had been hospitalized or had been a healthcare worker in a hospital outside of WA in the previous 12 months. MRSA-positive patients were isolated in the hospital, and staff with MRSA-positive test results received decolonization treatment. Imported MRSA still occasionally caused single-strain outbreaks in hospitals; however, infection control interventions contained them.

In the early 1990s, nonmultidrug-resistant MRSA (nmMRSA) were observed in WA, initially from indigenous people in remote communities (*7*) but subsequently in Perth, the state capital. These strains became known as "WA-MRSA." Although WA-MRSA did not readily spread in WA hospitals, 1 strain was responsible for an outbreak of hospital-acquired infection (*8*). Strains of nmMRSA have recently been reported in the eastern Australian states, and studies in Queensland and New South Wales showed a strong association between community-acquired infection with nmMRSA and Polynesian ethnicity. Isolates causing these infections were indistinguishable by phage typing and pulsed-field gel electrophoresis from those previously reported in New Zealand (*9**,**10*). Subsequently, a second strain (WA-MRSA-7 or Qld MRSA) has been associated with community-acquired infections in Caucasians in Queensland (*11*).

The emergence of nmMRSA has also been reported in other parts of the world, including North America (*12*) and Europe (*13*). Although nmMRSA strains appear to have originated in the community, they may include nmEMRSA strains that have been associated with healthcare facilities (e.g., EMRSA-15, EMRSA-16, and the New York/Japan EMRSA) or nonmultidrug-resistant sporadic hospital MRSA strains that have been taken into the community.

In 1997, the Department of Health WA, in collaboration with the Department of Microbiology and Infectious Diseases at Royal Perth Hospital, PathWest Laboratory Medicine WA, and the School of Biomedical Sciences at Curtin University of Technology established the Gram-positive Bacteria Typing and Research Unit to assist in controlling MRSA in WA. Since then, all MRSA isolated in WA have been referred to the unit for epidemiologic typing. This study describes the different epidemic and CA-MRSA clones isolated in WA and establishes their genetic relatedness.

## Materials and Methods

### MRSA Isolates

All MRSA isolated in WA between July 1, 2003, and December 31, 2004, were included in this study. Isolates were recovered from clinical and infection control screening specimens. For the purpose of this study, duplicate isolates from the same patient (as determined by their antimicrobial drug susceptibility phenotype) were excluded.

### Antimicrobial Susceptibility Testing

A test for oxacillin susceptibility was performed on Mueller-Hinton agar by the disk diffusion method according to Clinical Laboratory Standards Institute (CLSI) recommendations by using a 1-μg oxacillin disk (*14*). Oxacillin susceptibility results discrepant with those of the referring laboratory were confirmed by the detection of the *mecA* gene by polymerase chain reaction (PCR) (*15*).

An antibiogram was performed on Mueller-Hinton agar by the disk diffusion method according to CLSI recommendations, against a panel of 8 antimicrobial drugs (*14*): erythromycin (15 μg), tetracycline (30 μg), trimethoprim (5 μg), ciprofloxacin (5 μg), gentamicin (10 μg), rifampin (5 μg), fusidic acid (10 μg), and mupirocin (5 μg). The French CA-SFM susceptibility testing interpretive criterion was used for fusidic acid (*16*), and the suggested interpretive criterion by Finlay et al. was used for mupirocin (*17*). CLSI interpretive criteria were used for the remaining antimicrobial drugs (*18*). MRSA that were resistant to >3 of the 8 antimicrobial drugs listed were defined as mMRSA and those resistant to <3 drugs were defined as nmMRSA (*8*). Urease production was performed by Christensen's urea slopes incubated for 24 h at 37°C.

### Typing Methods

Resistogram typing was performed by disk diffusion against a panel of 6 chemicals and dyes: cadmium acetate (10 mmol/L), sodium arsenate (0.2 μmol/L), ethidium bromide (15 mmol/L), propamidine isethionate (2% [wt/vol]), mercuric chloride (0.4 μmol/L), and phenylmercuric acetate (5 mmol/L) as previously described (*19**,**20*).

Coagulase gene restriction fragment length polymorphism (RFLP) typing was performed as previously described (*21*). Contour-clamped homogeneous electric field electrophoresis (CHEF) was performed as previously described with the CHEF DR III System (Bio-Rad Laboratories Pty Ltd, Regents Park, New South Wales) (*8*). Chromosomal patterns were examined visually, scanned with a Fluor-S Multimager (Bio-Rad Laboratories), and digitally analyzed with Multi-Analyst/PC (Bio-Rad Laboratories). CHEF patterns were grouped according to the criteria of Tenover et al. (*22*) and using a dendrogram similarity of >80% to assign strain relatedness. *S. aureus* NCTC8325 was used as the size marker.

Multilocus sequence typing (MLST) was performed as specified by Enright et al. (*23*) on randomly selected isolates within each pulsotype. To assign a sequence type (ST) the sequences obtained were compared with the sequences described on the MLST website (http://www.mlst.net). Using the MLST database, clones were subsequently grouped into clonal complexes (CC).

SCC*mec* typing was performed by PCR by using previously published primers that identified the class of *mec* complex and type of cassette chromosome recombinase (*ccr*) encoded on the element (*24**,**25*). The presence of the Panton-Valentine leukocidin (PVL) determinants were detected by PCR by using previously published primers (*26*) and confirmed by sequencing the products.

## Results

A total of 4,099 MRSA isolates were studied. All isolates were initially grouped according to their antibiogram and urease production. Isolates within a group were then further characterized by using coagulase PCR-RFLP and CHEF analysis. MLST/SCC*mec* typing and PVL detection were performed on randomly selected isolates within each pulsotype. Twenty-nine clones were identified, including 7 (22.5%) EMRSA clones and 22 (77.5%) CA-MRSA clones.

### EMRSA Clones

Table 1 shows the 7 EMRSA clones identified: ST22-MRSA-IV (EMRSA-15), ST239-MRSA-III (Aus-2 and Aus-3 EMRSA), ST8-MRSA-IV_pediatric_ (Irish-2 EMRSA), ST36-MRSA-II (EMRSA-16), ST5-MRSA-II (New York/Japan EMRSA), ST8-MRSA-II_variant_ (Irish-1 EMRSA), and the classic MRSA clone ST250-MRSA-I. Only 3 EMRSA clones were typically multidrug-resistant: ST239-MRSA-III (resistant to tetracycline, erythromycin, trimethoprim, ciprofloxacin, and gentamicin), ST8-MRSA-IV_pediatric_ (resistant to erythromycin, trimethoprim, and ciprofloxacin), and ST8-MRSA-II_variant_ (resistant to tetracycline, erythromycin, trimethoprim, ciprofloxacin, gentamicin and mupirocin).

**Table 1:** Characteristics of EMRSA clones* (of 4,099 total MRSA isolates), Western Australia, July 1, 2003–December 31, 2004

Clone	CHEF pattern (pulsotypes)	n (% of total MRSA)	CC	Urease	Coagulase PCR RFLP pattern	PVL toxin
ST22-MRSA-IV	EMRSA-15	719 (17.54)	22	Neg	22	Neg
ST239-MRSA-III	Aus-2 EMRSA	95 (2.32)	8	Pos	24	Neg
	Aus-3 EMRSA	57 (1.39)	8	Pos	24	Neg
ST8-MRSA-IVp	Irish-2 EMRSA	20 (0.49)	8	Neg	18	Neg
ST36-MRSA-II	EMRSA-16	16 (0.39)	30	Pos	18	Neg
ST5-MRSA-II	New York/Japan EMRSA	11 (0.27)	5	Pos	36	Neg
ST8-MRSA-IIv	Irish-1 EMRSA	2 (0.05)	8	Neg	18	Neg
ST250-MRSA-I	Classic MRSA	1 (0.02)	8	Pos	18	Neg
Total		921 (22.47)				

*EMRSA, epidemic methicillin-resistant *Staphylococcus aureus*; CHEF, contour-clamped homogeneous electric field; CC, clonal complex; PCR, polymerase chain reaction; RFLP, restriction fragment length polymorphisms; PVL, Panton-Valentine leukocidin; MRSA, methicillin-resistant *S. aureus;* p, pediatric; v, variant.

Overall, 94.6% of EMRSA were identified either as ST22-MRSA-IV (78.1%), a urease-negative nmEMRSA clone (resistant to erythromycin and ciprofloxacin) or ST239-MRSA-III (16.5%). By using CHEF electrophoresis and resistogram typing, ST239-MRSA-III could be further classified into 2 subclones: Aus-2 EMRSA (susceptible to mercuric chloride and phenylmercuric acetate) and Aus-3 EMRSA (resistant to mercuric chloride and phenylmercuric acetate).

### CA-MRSA

Of the 22 identified clones of CA-MRSA, 21 were WA-MRSA and 1 was Western Samoan phage pattern (WSPP) MRSA (Table 2). By using CHEF electrophoresis, ST8-MRSA-IV and ST5-MRSA-V could be further classified into WA-MRSA-5 and WA-MRSA-12, and WA-MRSA-11 and WA-MRSA-14 pulsotypes, respectively.

**Table 2:** Characteristics of community MRSA clones* (of 4,099 total MRSA isolates), Western Australia, July 1, 2003–December 31, 2004

Clone	CHEF pattern (pulsotypes)	n (% of total MRSA)	CC	Urease	Coagulase PCR RFLP pattern	PVL toxin
ST1-MRSA-IV	WA MRSA-1	1,757 (42.86)	1	Pos	20	Neg
ST129-MRSA-IV	WA MRSA-2	947 (23.10)	78	Pos	258	Neg
ST5-MRSA-IV	WA MRSA-3	273 (6.66)	5	Pos	36	Neg
ST45-MRSA-V	WA MRSA-4	60 (1.46)	45	Pos	DNC	Neg
ST8-MRSA-IV	WA MRSA-5	27 (0.66)	8	Pos	18	Neg
	WA MRSA-12	4 (0.1)	8	Pos	18	Pos
ST93-MRSA-IV	WA MRSA-7	34 (0.83)	S	Pos	32	Pos
ST75-MRSA-IV	WA MRSA-8	9 (0.22)	S	Pos	DNA	Neg
ST59-MRSA-V	WA MRSA-9	3 (0.07)	59	Pos	40	Pos
ST573-MRSA-V	WA MRSA-10	2 (0.05)	1	Pos	34	Neg
ST5-MRSA-V	WA MRSA-11	11 (0.27)	5	Pos	34	Neg
	WA MRSA-14	4 (0.09)	5	Pos	40	Neg
ST584-MRSA-IV	WA MRSA-13	1 (0.2)	9	Pos	32	Neg
ST59-MRSA-IV	WA MRSA-15	3 (0.07)	59	Pos	40	Neg
ST8-MRSA-Novel	WA MRSA-16	1 (0.2)	8	Neg	18	Neg
ST583-MRSA-IV	WA MRSA-17	2 (0.5)	80	Pos	DNC	Pos
ST5-MRSA-Novel	WA MRSA-18	1 (0.02)	5	Pos	36	Neg
ST609-MRSA-IV	WA MRSA-19	1 (0.02)	8	Neg	18	Neg
ST5-MRSA-Novel	WA MRSA-21	2 (0.5)	5	Pos	34	Neg
ST577-MRSA-V	WA MRSA-22	3 (0.07)	121	Pos	42	Neg
ST45-MRSA-IV	WA MRSA-23	1 (0.02)	45	Neg	22	Neg
ST87-MRSA-IV	WA MRSA-24	1 (0.02)	59	Pos	40	Neg
ST575-MRSA-IV	WA MRSA-25	1 (0.02)	5	Pos	256	Neg
ST30-MRSA-IV	WSPP MRSA	30 (0.73)	30	Pos	24	Pos
Total		3,178 (77.53)				

*MRSA, methicillin-resistant *Staphylococcus aureus*; CHEF, contour-clamped homogeneous electric field; CC, clonal complex; PCR, polymerase chain reaction; RFLP, restriction fragment length polymorphisms; PVL, Panton-Valentine leukocidin; WA MRSA, Western Australia MRSA; DNC, did not cut; S, singleton; DNA, did not amplify; WSPP MRSA, Western Samoan phage pattern MRSA.

Overall, 93.7% of CA-MRSA were classified into 3 clones: ST1-MRSA-IV (55.3%), ST129-MRSA-IV (29.8%), and ST5-MRSA-IV (8.6%). Of the CA-MRSA, 97.3% were SCC*mec* type IV and 2.6% SCC*mec* type V. Four isolates carrying novel SCC*mec* type(s) were found in 2 STs (ST5 and ST8). Using the MLST database, the 22 clones were grouped into 10 CCs and 2 singletons.

Five clones (2.3% of CA-MRSA) were PVL positive including ST30-MRSA-IV and ST93-MRSA-IV. These 2 clones were originally reported outside WA, ST30-MRSA-IV in New Zealand and ST93-MRSA-IV in Queensland. The remaining 3 clones, ST8-MRSA-IV (MRSA-12 pulsotype), ST59-MRSA-IV, and ST583-MRSA-IV accounted for only 0.3% of CA-MRSA isolated in WA.

### CA-MRSA Antibiograms

Table A1 shows 6 of the 22 CA-MRSA clones (17 isolates) were predictably resistant to β-lactam antimicrobial drugs only. In the remaining 16 clones, 55.9% of isolates were resistant to at least 1 non–β-lactam antimicrobial drug, including 47.7% to erythromycin, 10.8% to fusidic acid, 2.9% to ciprofloxacin, 2.5% to trimethoprim, 1.8% to tetracycline, 1.6% to gentamicin, 1.3% to mupirocin, and 0.2% to rifampin. In 6 of these clones, 1.5% of isolates were classified as multidrug-resistant. None of the 73 isolates found in PVL-positive clones were multidrug-resistant: 7% of ST30-MRSA-IV were resistant to rifampin, 15% of ST93-MRSA-IV were resistant to erythromycin, 50% of ST583-MRSA-IV were resistant to fusidic acid and tetracycline, and 50% were resistant to fusidic acid and erythromycin; all the ST59-MRSA-V were resistant to tetracycline and erythromycin, and 75% and 25% of ST8-MRSA-IV (MRSA-12 pulsotype) were resistant to erythromycin and tetracycline, respectively. The 11 strains classified as ST5-MRSA-V (WA-MRSA-11 pulsotype) were all resistant to gentamicin. This strain was involved in a single strain outbreak in a burn unit.

## Discussion

In WA, colonization or infection with MRSA has been a notifiable condition since 1982, which has enabled the rapid and widespread emergence of CA-MRSA to be monitored. In rural areas, the overall MRSA notification rate has increased from 10/100,000 persons in 1983 to 542/100,000 persons in 2002. Similarly, in the same period rates increased in the Perth metropolitan area from 7/100,000 to 520/100,000. Although part of this increase in the metropolitan area from 1998 was due to an increase in EMRSA notifications, most can be attributed to CA-MRSA (unpub. data).

In this study, 22.5% (n = 921) of MRSA isolates were classified as EMRSA. Six international epidemic clones and the classic MRSA clone, ST250-MRSA-I, were identified (Table 1). More than 78% of EMRSA isolates were identified as ST22-MRSA-IV (EMRSA-15), an nmEMRSA with the community type IV SCC*mec*. Originally described in the United Kingdom in the early 1990s and now the predominant epidemic strain in that country (*27*), ST22-MRSA-IV was first isolated in WA in 1997 in preemployment screening of healthcare workers coming from the United Kingdom and Ireland (*28*). Notifications have increased from 55 in 1998 to 383 in 2002 (unpub. data). Although recent national surveillance studies have also reported the emergence of ST22-MRSA-IV in other Australian states (*29*), the predominant EMRSA in most Australian capital cities is ST239-MRSA-III (*30*). The 6 epidemic clones reported in this study have 6 STs which can be grouped into 4 of the 5 major epidemic CCs described by Enright et al. (*5*), CC5, CC8, CC22, and CC30. CC8 also includes the ancestral MRSA genotype ST250-MRSA-I, which evolved from the methicillin-susceptible strain ST250-MSSA, which is thought to have arisen from ST8-MSSA by a chromosomal mutation (*5*). Other STs isolated in this study, which form part of CC8, were ST8-MRSA-IV_pediatric_, ST8-MRSA-II_variant_, and ST239-MRSA-III. ST5-MRSA-II, ST22-MRSA-IV, and ST36-MRSA-II belong to CC5, CC22, and CC30, respectively.

CA-MRSA made up 77.5% of the isolates. These MRSA have several characteristics that differentiate them from most nosocomial MRSA. They harbor a smaller, SCC*mec* (SCC*mec* IV and V), are susceptible to most antimicrobial drugs other than the β-lactam agents, and are more likely to encode the virulence factor PVL (*31*).

In this study, 21 clones of WA-MRSA were identified by MLST/SCC*mec* typing with further delineation into 23 chromosomal DNA pulsotypes and numerous pulsosubtypes by CHEF. Also identified was the Western Pacific CA-MRSA (ST30-MRSA-IV) first isolated in Auckland, New Zealand (*32*). CA-MRSA from different parts of the world has been reported with varied genetic backgrounds (*24*). The results presented here demonstrate that this is also the case for CA-MRSA isolated within a single state of Australia (WA). The 22 STs were grouped into 10 CCs (CC1, CC5, CC8, CC9, CC30, CC45, CC59, CC78, CC80, and CC121) and 2 singletons (ST75-MRSA-IV and ST93-MRSA-IV). The 10 CCs identified include 4 of the 5 major epidemic CCs (CC5, CC8, CC30, and CC45). Five CCs had >1 clone (2 clones in CC1 and CC45, 3 clones in CC8 and CC59, and 5 clones in CC5).

The 2 predominant CA-MRSA clones isolated were ST1-MRSA-IV and ST129-MRSA-IV (55.3% and 29.8% of CA-MRSA, respectively). ST1-MRSA-IV belongs to CC1, which has the same allelic profile as the *S. aureus* that is the proposed ancestor of MW2 CA-MRSA that was responsible for the deaths of 4 children in the United States (*33*). CC1 CA-MRSA has also been reported in France (*13*) and other areas of Australia (*24*), which indicates that this clone is particularly successful. ST129-MRSA-IV belongs to CC78, a smaller CC that includes strains isolated elsewhere in Australia, Portugal, and Japan (http://www.mlst.net).

Despite the diversity of CCs, the CA-MRSA strains were remarkably uniform in their SCC*mec* allotypes. SCC*mec* IV was identified in 14 STs and SCC*mec* V was identified in 5. This suggests that in WA these 2 allotypes are well adapted to the community environment. Two STs were found to have novel SCC*mec* types.

Unlike SCC*mec* types II and III, which carry a number of inserted plasmids and transposons downstream of the *mecA* complex, community-associated SCC*mec* types IV and V are smaller and lack other resistance genes. However, resistance may be encoded elsewhere on the chromosome or the isolate may carry resistance plasmids. Although CA-MRSA isolated in WA is typically nonmultidrug-resistant, all strains harbor a large plasmid that varies in size (*34*). This plasmid encodes determinants for β-lactamase production and cadmium resistance. In addition, some isolates have been reported to carry a 41.4-kb plasmid that also encodes β-lactamase and resistance to mupirocin, tetracycline, trimethoprim, and cadmium and a smaller plasmid (2 kb) that encodes inducible erythromycin resistance (*34*). Chromosomal fusidic acid and tetracycline resistance determinants have also been reported (*34*); however, the location of these determinants on the chromosome is unknown. In this study, 44% of CA-MRSA were resistant to β-lactam antimicrobial drugs only. Of the remaining 56%, 54.5% were also resistant to 1–2 non–β-lactam agents, and 1.5% to >3 non–β-lactam agents, including 3 isolates resistant to 5 additional antimicrobial drugs (Table A1).

CA-MRSA have been shown to express several virulence genes, including the determinants for PVL (*35*). PVL is a necrotizing toxin that causes leukocyte destruction and tissue necrosis and is associated with abscesses and severe pneumonia. PVL is present in most of the CA-MRSA studied in Europe and the United States (*13*). In WA, CA-MRSA infrequently carries the genes encoding PVL (*34*); however, 2 CA-MRSA clones, ST30-MRSA-IV, and ST93-MRSA-IV, more commonly isolated in eastern Australia are PVL positive. ST30-MRSA-IV was first noted in Australia in 1997 in the Polynesian population living in the eastern Australian states and the Australian Capital Territory (*9*). ST93-MRSA-IV was first identified as a cause of community-acquired infection in the Caucasian population in Ipswich, Queensland, in 2000 (*11*). Both clones are now frequently isolated in several areas of Australia (*29*). In WA, ST30-MRSA-IV and ST93-MRSA-IV were first isolated in 2001. In this study, PVL was detected in 5 MRSA clones, including ST30-MRSA-IV, ST93-MRSA-IV, ST8-MRSA-IV (pulsotype WA-MRSA-12), ST59-MRSA-V, and ST583-MRSA-IV. However, these 5 clones were infrequently isolated and accounted for only 2.3% of all CA-MRSA. PVL genes have been transmitted by a temperate phage designated φPVL (*36*), which indicates that the PVL determinants are transferable. Recently, a PVL-positive ST1-MRSA-IV strain was isolated in Queensland (*37*) and New South Wales (*38*), Australian states that have reported an increasing incidence of ST30-MRSA-IV and ST93-MRSA-IV (*9**–**11*). This finding suggests that the PVL determinants are being transferred and raises the prospect that more CA-MRSA in WA may acquire PVL determinants in the future.

Some researchers have proposed that CA-MRSA may arise either by hospital strains escaping into the community, where they spread person to person, or de novo when the SCC*mec* complex is acquired by a methicillin-susceptible *S. aureus* isolate (*24**,**39*). In WA, the genetic background of nosocomial MRSA is different from that of CA-MRSA, and therefore, community strains have likely evolved independently of hospital strains. In addition, in WA hospitals, apart from 2 single-strain outbreaks in a large metropolitan hospital (ST1-MRSA-IV [13] and ST5-MRSA-V (WA-MRSA-11 pulsotype), little evidence has been found of CA-MRSA spreading within healthcare facilities. Although person-to-person spread most likely occurs in the community, the increasing number of MRSA in the WA community may also be due to mobility of the community SCC*mec* types. The genetic diversity of CA-MRSA isolated in WA and the presence of at least 3 SCC*mec* types also support this possibility.

## Conclusions

Although a comprehensive MRSA screening and control program has prevented the mEMRSA from emerging, it has not prevented SCC*mec* type IV and type V MRSA clones, including nmEMRSA (ST22-MRSA-IV) and CA-MRSA, from becoming established in WA. SCC*mec* types IV and V are now found in MRSA with distantly related genetic backgrounds. In addition, at least 1 novel SCC*mec* type has been detected. Initially nonmultidrug-resistant, many of these CA-MRSA clones have acquired plasmids and chromosomal resistance determinants allowing some strains to become resistant to up to 5 non–β-lactam antimicrobial agents, including erythromycin, tetracycline, trimethoprim, ciprofloxacin, gentamicin, rifampin, fusidic acid, and mupirocin. With the detection of 5 PVL-positive clones and the recent emergence of PVL in a previously reported PVL-negative CA-MRSA clone, more severe staphylococcal disease caused by CA-MRSA can be expected in the future. SCC*mec* types that can be acquired by multiple genotypes of *S. aureus* over a short period and the isolation of multidrug-resistant or PVL-positive CA-MRSA are major public health concerns and emphasize the importance of typing in tracing the origin of isolates and in designing antimicrobial drug prescribing policies for their control, if possible, in the community.

## Appendix

**Table A1:** Community MRSA antibiogram*. Download as PDF [61 KB, 3 pages].

	ST 1 M R S A IV	ST 129 M R S A IV	ST 5 M R S A IV	ST 45 M R S A V	ST 8 M R S A IV	ST 93 M R S A IV	ST 75 M R S A IV	ST 59 M R S A V	ST 573 M R S A V	ST 5 M R S A V	ST 584 M R S A IV	ST 59 M R S A IV	ST 8 M R S A nov	ST 583 M R S A IV	ST 5 M R S A nov	ST 609 M R S A IV	ST 5 M R S A nov	ST 577 M R S A V	ST 45 M R S A IV	ST 87 M R S A IV	ST 575 M R S A IV	ST 30 M R S A IV	T O T A L	%
β-lactam resistance only (44.05%)																								
O	1107	16	139	53	11	29	9					3			1		2		1		1	28	1400	44.05
β-lactam plus 1 non– β-lactam antimicrobial drug resistance (45.06%)																								
O, E	213	883	87	4	4	5					1							3		1			1201	37.79
O, F	132	1	3	1					2														139	4.37
O, C	29		4	1	1																		35	1.10
O, Tp	17	1	3		4																		25	0.79
O, Tc	10		1	1	1																		13	0.41
O, G			2							11													13	0.41
O, M	3																						3	0.09
O, R	1																					2	3	0.09
β-lactam plus 2 non–β-lactam antimicrobial drug resistance (9.44%)																								
O, E, F	179	4	1											1									185	5.82
O, E, C	3	18	16		6																		43	1.35
O, E, Tc	17	3			1			3															24	0.76
O, E, Tp	1	14																					15	0.47
O, G, Tp	8		2																				10	0.31
O, Tp, M	5		2																				7	0.22
O, E, R		5																					5	0.16
O, G, M	1		3																				4	0.13
O, E, M	1	1																					2	0.06
O, T, F	1													1									2	0.06
O, G, F	1																						1	0.03
O, G, C	1																						1	0.03
O, C, M													1										1	0.03
β-lactam plus 3 non–β-lactam antimicrobial drug resistance (0.98%)																								
O, E, TpR G	9		1		1																		11	0.35
O, E, G, M			4																				4	0.13
O, E, C, F	3																						3	0.09
O, Tc, F, M	1									2													3	0.09
O, E, Tp, M,	2	1																					3	0.09
O, E, C, M			1		1																		2	0.06
O, E, Tc, M,	1																						1	0.03
O, E, C, Tc			1																				1	0.03
O, E, C, Tp			1																				1	0.03
O, Tp, G, M	1																						1	0.03
O, Tc, G, M	1																						1	0.03
β-lactam plus 4 non–β-lactam antimicrobial drug resistance (0.38%)																								
O, E, Tc, F, M	6									1													7	0.22
O, E, C, Tc, G			2																				2	0.06
O, E, Tc, F, Tp										1													1	0.03
O, E, Tp, G, R					1																		1	0.03
O, E, F, M, G	1																						1	0.03
β-lactam plus 5 non–β-lactam antimicrobial drug resistance (0.09%)																								
O, E, C, F, M, G																1							1	0.03
O, E, C, F, M, Tp	1																						1	0.03
O, E, C, F, Tc, Tp	1																						1	0.03
TOTAL	1757	947	273	60	31	34	9	3	2	15	1	3	1	2	1	1	2	3	1	1	1	30	3178	

*MRSA, methicillin-resistant *Staphylococcus aureus*; O, oxacillin; Tc, tetracycline; E, erythromycin; Tp, trimethoprim; C, ciprofloxacin; G, gentamicin; F, fusidic acid; R, rifampin; M, mupirocin; nov, novel.
